# Olfaction and Gustation in Children With Primary Ciliary Dyskinesia

**DOI:** 10.1002/oto2.28

**Published:** 2023-02-17

**Authors:** Faisal Zawawi, Sharon Dell, Nikolaus E. Wolter, Blake C. Papsin, Evan J. Propst

**Affiliations:** ^1^ Department of Otolaryngology–Head and Neck Surgery Hospital for Sick Children Toronto Ontario Canada; ^2^ Department of Otolaryngology–Head and Neck Surgery King Abdulaziz University Jeddah Saudi Arabia; ^3^ Department of Pediatrics, Division of Respiratory Medicine, BC Children's Hospital University of British Columbia Vancouver British Columbia Canada

**Keywords:** children, gustation, olfaction, primary ciliary dyskinesia, U‐Sniff

## Abstract

**Objective:**

Primary ciliary dyskinesia (PCD) is a rare autosomal recessive disorder whereby abnormal cilia cause a wide array of respiratory tract manifestations including chronic rhinosinusitis. The purpose of this study was to determine whether olfaction and gustation are impaired in children with PCD.

**Study Design:**

Cross‐sectional study.

**Setting:**

Tertiary pediatric academic hospital.

**Methods:**

Children with confirmed PCD based on having at least 1 of 3 approved diagnostic criteria as per The American Thoracic Society guidelines were recruited from The PCD Clinic in our tertiary care pediatric hospital. Odor identification ability was tested using the Universal Sniff (U‐Sniff) test and taste threshold was measured using an electrogustometer. The main outcome of this study is to determine the incidence of olfactory dysfunction in children with PCD and investigate if there is an associated gustatory dysfunction.

**Results:**

Twenty‐five children participated (14 male, 11 female), The median age was 10.8 years (range: 4.1‐17.9 years). Only 4/25 (16%) complained of olfactory dysfunction prior to testing. None of the patients complained of dysgeusia. However, 48% (12/25) scored less than 7 on the U‐Sniff, signifying hyposmia or anosmia. In contrast, scores obtained by electrogustometry were in the normal range. There was no correlation between performance on the U‐Sniff and electrogustometry testing.

**Conclusion:**

Olfactory impairment in children with PCD is common but underrecognized by patients. This is not associated with abnormal gustation. Among other, this places children with PCD at an increased risk with respect to smelling a fire or detecting spoiled or poisonous food.

Primary ciliary dyskinesia (PCD) is a rare, primarily autosomal recessive disorder, with a prevalence of 1 in 15,000 to 30,000.^1^ In PCD, mutations in over 40 different genes result in the abnormal structure and function of respiratory cilia.^1^ These defects lead to reduced mucociliary clearance, resulting in chronic upper and lower respiratory tract infections. Most patients with PCD experience a year‐round wet cough and nasal congestion from early infancy, neonatal respiratory distress despite being born at term, and chronic bronchitis which eventually progresses to bronchiectasis and often respiratory failure.^1^, ^2^ However, PCD is often missed in children because it is rare, the presentation is similar to recurrent viral respiratory illness, and confirmation requires specialized diagnostic testing such as transmission electron microscopy, costly genetic panels, or nasal nitric oxide (nNO) testing.^3^

Primary cilia have been shown to be involved in olfaction in transgenic mice and patients with Bardet‐Biedl syndrome.^4^ In these patients, olfactory deficits are primarily due to dysfunction of the basal bodies or cilia.^4^ In addition, many children with PCD have chronic rhinosinusitis and suffer from significant nasal obstruction, both of which can be associated with olfactory impairment.^2^, ^5^, ^6^, ^7^, ^8^

A study by our group looking into otolaryngology‐related manifestations reported that some of the children with PCD complained of anosmia.^9^ Furthermore, a recently published study noted the presence of olfactory dysfunction in children with PCD.^10^ Nevertheless, olfactory dysfunction is still an underinvestigated phenotype of PCD. Additionally, the relationship between olfactory function and its relation to the sense of taste in children with PCD has never been reported, despite of the high interconnectivity between the 2 chemical senses.^11^ Hence, the objective of this study was to identify the prevalence of olfaction dysfunction in children with PCD and to determine its association with taste.

## Materials and Methods

This study was approved by the Hospital for Sick Children ethical review board (protocol number 1000028062). Children were recruited from the Respiratory Medicine PCD clinic at our large tertiary care pediatric center. Parents of children 4 years of age and older were approached for recruitment. With children assenting informed consent about the objective of this study, protocol and details was then obtained from the participants and/or /parents/legal guardian of the participant. The diagnosis of PCD was based on the PCD guidelines. All recruited participants were diagnosed with PCD through at least 1 of 3 approved diagnostic criteria as per The American Thoracic Society guidelines^9^, ^12^, ^13^: (1) 2 pathogenic or likely pathogenic mutations in 1 known PCD gene, (2) Presence of a disease‐causing ciliary ultrastructural defect on transmission electron microscopy (TEM, including outer dynein arm defect, outer plus inner dynein arm defect, or absent inner dynein arm with central apparatus defect and microtubule disorganization), or (3) nNO values <77 nL/min, by exhalation against resistance, on at least 2 separate occasions, with cystic fibrosis ruled out via sweat chloride or cystic fibrosis transmembrane conductance regulator genetic testing in children ≥5 years old.^7^, ^12^ Participants older than 18 years at the time of recruitment were excluded.

After written informed consent was obtained from the parents (or the legal guardian of the child), each child had clinical history documented and physical examination performed including flexible nasopharyngolaryngoscopy (all of which were performed by the same pediatric otolaryngologist “FZ”), to assess for features of chronic rhinosinusitis. Participants deemed not to be at their baseline functioning, for example, with an upper or lower respiratory tract infection, had their testing delayed for 8 weeks. Demographic and clinical data, including specific genetic PCD mutation, were collected. Sinonasal symptoms and signs were recorded on a standardized form. Participants were administered the 22‐item questionnaire (SNOT‐22) to determine the burden of their sinonasal symptoms.^14^

### Olfaction and Gustation

Olfaction was tested using the Universal Sniff (U‐Sniff) test (Burhart), an international odor identification test for children. This test has been validated for children age 3 to 18 years.^15^ During the U‐Sniff, children are presented with 12 olfactory stimuli and must select what each odor represents from a series of 4 photographs/verbal items. A total score of less than 7 is considered abnormal based on a study that included our institution.^15^ Taste threshold testing was measured using an electrogustometer (Rion TR‐06; Rion Co. Ltd). This electrogustometer has previously been used at our institution to assess taste in children.^16^ As with other sensory systems, the taste responses can be expressed on a logarithmic scale and hence the RION TR06 has a current control which operates with logarithmic steps labeled as decibels. The scale is calibrated to make 0 dB = 8 μA and hence the total range is −6 to +34 dB according to the formula dB = log_10_[μA/8]. The calculated normal range is −6 to 2. Anything higher than 2 was considered abnormal.

### Data Analysis

Data were collected using Microsoft Excel v 14.7.7 (Microsoft) and analyzed using SPSS v24 (IBM). Nonparametric *χ* ^2^ tests were used for categorical data and Mann‐Whitney *U*‐tests for continuous data. A *p*‐value of less than .05 was considered significant.

## Results

### Demographics and Genetics

A total of 25 participants were recruited for this study. The median age was 10.8 years (range: 4.1‐17.9 years, interquartile range [IQR] = 8.88). The most common genetic mutations were DNAH5 8/25 (32.2%), DNAH11 4/25 (16%) (10.6%), and CCNO 3/25 (12%). Four participants (16%) were negative or refused genetic testing but were phenotypically confirmed based on the American Thoracic Society Clinical Practice Guidelines (Table 1). The majority of the children with PCD (19/25) in this cohort were not followed up regularly with an otolaryngologist. Pre‐existing regular sinonasal medical management included: combination of topical nasal saline and steroids sprays (3/25), nasal saline sprays alone (5/25), and nasal steroid sprays alone (1/25).

**Table 1:** Various Genetic Mutations of Children With Normal U‐Sniff Results and Abnormal U‐Sniff Results

Olfactory function	Gene mutation	N (25)
Normal U‐Sniff score	DNAH5	5
	DNAH11	3
	CCDC40	2
	CCNO	1
	Negative (phenotypically confirmed)	3
Abnormal U‐Sniff score	DNAH5	3
	CCNO	2
	ARMC4	1
	DNAH11	1
	CCDC114	1
	CCDC39	1
	DNAI1	1
	Negative (phenotypically confirmed)	1

Abbreviation: U‐Sniff, Universal Sniff.

### Olfaction and Gustation Testing

All 25 children clinically met the criteria for chronic rhinosinusitis based on history and clinical examination and endoscopy features that were consistent with the clinical practice guidelines for the American Academy of Otolaryngology–Head and Neck Surgery and the American Association of Pediatrics clinical practice. practice.^17^, ^18^ Prior to olfactory testing, participants and their parents were asked if they had a problem with their sense of smell. Only 4/25 (16%) reported suspicion of change in smell or inability to smell. The median SNOT‐22 score was 38 (range: 2‐91, IQR = 38.5).

All 25 children were able to complete the testing. Overall, 13/25 (52%) of participants scored less than 7 on the U‐Sniff odor identification test, which is considered abnormal (median U‐Sniff = 6, IQR = 6). All participating children had normal electrogustometry thresholds. The median electrogustometry threshold score was −4 (IQR = 2). There was no correlation between U‐Sniff and electrogustometry scores (Pearson's correlation = .08, *p* = .70). The median (IQR) SNOT‐22 score for children was 38 (IQR = 38.5), and there was no statistical difference in the median SNOT‐22 score of children who had anosmia and those who were normosomic (38 and 48, respectively, *p* > .05). There was no major difference in the prevalence of anosmia across mutation type (*p* > .05).

**Figure 1:**
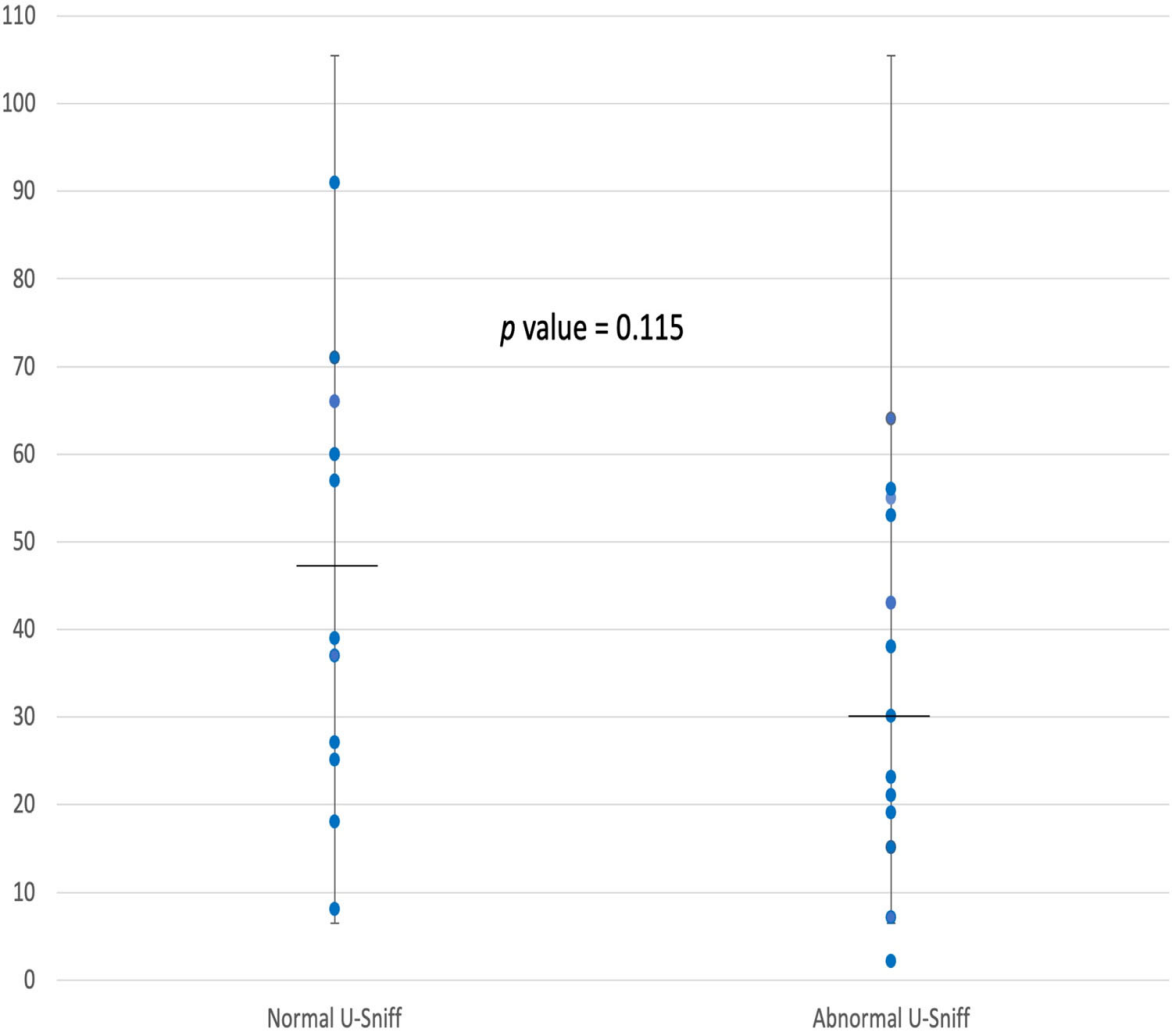
This scattered plot demonstrates the SNOT‐22 scores for the group with abnormal Universal Sniff (U‐Sniff) results and normal U‐Sniff result. The horizontal line represents the median score.

The majority of children with PCD were not using any sinonasal topical treatment regularly (16/25), there were 4 children who were using topical nasal steroids therapy regularly (1 alone and 3 in combination with saline irrigation). Out of those 4 children, only 1 had anosmia on U‐Sniff (Figure 1).

## Discussion

There is a paucity of data about olfaction in children with PCD, and only recently 2 publications have discussed the possibility of olfactory dysfunction in children with PCD. The present study identified the prevalence of olfactory dysfunction in children with PCD and determined its association with taste. We tested 25 children with PCD using objective olfactory and gustatory testing. More than one‐half of children with PCD in this study (52%) had abnormal olfaction using the U‐Sniff test, the majority (84%) of whom were not aware of this deficiency.

Altered sense of smell is one of the more frequent complaints from patients with chronic rhinosinusitis,^19^ and 100% of children in our study had chronic rhinosinusitis. In a recently published study, 29% of children with PCD had anosmia when tested with Sniffin' Sticks. The study also adds that another 45% of the children had hyposmia.^10^ This is in keeping with the results found in our cohort. Awareness of a deficiency often relies on recall of normal function at an earlier time as well as if it was gradually lost (eg, in the aging population or those with chronic sinonasal disease).^20^ It is possible that the majority of patients with PCD never had a normal olfactory function, making a comparison with that time impossible. Additionally, most people are not very good at rating the quality and strength of their olfactory function.^21^ Olfactory function contributes to the quality of life, and is essential for detecting hazardous materials such as a gas leak or rotten food.^22^ Results from this study suggest that the majority of children with PCD are at increased risk and should be carefully monitored with adjunct methods of detection created where possible.

Children with PCD benefit from multidisciplinary care.^7^ The majority of the children in this study were not regularly following up with otolaryngologist, nor where they receiving adequate treatment for chronic rhinosinusitis. In this population, only 4/25 (16%) were following up regularly with otolaryngologist, and despite the high incidence of chronic sinusitis in the cohort, only 3/25 (12%) were using both nasal steroids and saline sprays. Considering the possible changes of quality of life in relation to a normalized sense of smell,^23^ it appears to be necessary to counsel children with PCD and their families on their olfactory function.

There was no deficit in gustatory sensation in children with PCD and there was no association between gustatory thresholds and scores on the U‐Sniff. This suggests that taste is largely independent on the ability to have intact olfaction, at least in this population. However, using different techniques of measurement of gustatory functions other studies showed that taste is in fact decreased in relation to olfactory dysfunction, although this appears to be less pronounced in patients with sinonasal diseases.^24^

Regarding the genetic characterization of the children, the number of cases was too small to draw conclusions. Still, it might be interesting to note that 2 of the 3 children with a CCDC39 or CCDC40 mutation had a normal olfactory function. This differs from research in pulmonology, whereby children with CCDC39 and CCDC40 mutations tended to have a worse pulmonary disease burden.^7^

The mechanism behind olfactory dysfunction is not well understood. Pifferi et al^10^ questioned if the olfactory dysfunction is neural in origin similar to what is seen in primary ciliopathies. Although, olfactory dysfunction is a primary symptom of chronic rhinosinusitis mostly mediated by the inflammatory process,^25^ it is still unclear if anosmia in children with PCD has the same pathophysiology.

The limitations of this study are the small sample size which limits genotype‐phenotype analysis. Imaging, namely computed tomography scan, was not available for the majority of the patients, which made us unable to determine if the relationship between sinus disease burden with the olfactory dysfunction. Future studies should look into the effect of sinusitis treatment on olfactory dysfunction in children with PCD.

## Conclusion

Olfactory impairment in children with PCD is common and underrecognized. It does not seem to be associated with abnormal gustatory sensitivity. Anosmia in children with PCD may place them at increased risk due to their inability to smell a fire or spoiled or poisonous food and can lead to a decreased quality of life or depression. This should be considered when counseling and managing children with PCD.

## Author Contributions

**Faisal Zawawi**, conceptualized and designed the study, designed the data collection instruments, coordinated and supervised data collection, drafted the initial manuscript, and reviewed and revised the manuscript; **Sharon Dell**, coordinated and supervised data collection, and critically reviewed the manuscript for important intellectual content; **Nikolaus E. Wolter**, coordinated and supervised data collection, and critically reviewed the manuscript for important intellectual content; **Blake C. Papsin**, coordinated and supervised data collection, and critically reviewed the manuscript for important intellectual content; **Evan J. Propst**, conceptualized and designed the study, designed the data collection instruments, coordinated and supervised data collection, drafted the initial manuscript, and reviewed and revised the manuscript. All authors approved the final manuscript as submitted and agree to be accountable for all aspects of the work.

## Disclosures

### Competing interests

The authors have no conflicts of interest relevant to this article to disclose.

### Sponsorships

This project did not receive any external funding and was not sponsored commercial initity.

### Funding source

The authors have no financial relationships relevant to this article to disclose. No funding was necessary for this project.
